# Case Report: Takotsubo Syndrome Associated With Novel Coronavirus Disease 2019

**DOI:** 10.3389/fcvm.2021.614562

**Published:** 2021-02-22

**Authors:** Sofia Ortuno, Mathieu Jozwiak, Jean-Paul Mira, Lee S. Nguyen

**Affiliations:** ^1^Assistance Publique – Hôpitaux de Paris, Centre Cochin University Hospital, Intensive Care Medicine Department, Paris, France; ^2^Centre Médico-Chirurgical Ambroise Paré, Research and Innovation, Research and Innovation of CMC Ambroise Paré, Neuilly-sur-Seine, France

**Keywords:** Tako-tsubo cardiomyopathy, COVID−19, heart failure, acute respiratory distress syndrome, sepsis

## Abstract

**Background:** Takotsubo cardiomyopathy is triggered by emotional or physical stress. It is defined as a reversible myocardial dysfunction, usually with apical ballooning aspect due to apical akinesia associated with hyperkinetic basal left ventricular contraction. Described in cases of viral infections such as influenza, only few have been reported associated with novel coronavirus disease 2019 (COVID-19) in the recent pandemic.

**Case summary:** A 79-years-old man, with cardiovascular risk factors (type 2 diabetes and hypertension) and chronic kidney disease, presented to the emergency room for severe dyspnea after 8 days of presenting respiratory symptoms and fever. Baseline electrocardiogram (ECG) was normal, but he presented marked inflammatory syndrome. He was transferred to an intensive care unit to receive mechanical ventilation within 6 h, due to acute respiratory distress syndrome. He presented circulatory failure 2 days after, requiring norepinephrine support (up to up to 1.04 μg/kg/min). Troponin T was elevated (637 ng/l). ECG showed diffuse T wave inversion. Echocardiography showed reduced left ventricular ejection fraction (LVEF 40%), with visual signs of Takotsubo cardiomyopathy. Cardiac failure resolved after 24 h with troponin T decrease (433 ng/l) and restoration of cardiac function (LVEF 60% with regression of Takotsubo features). Patient died after 15 days of ICU admission, due to septic shock from ventilator-acquired pneumonia. Cardiac function was then normal.

**Conclusion:** Mechanisms of Takotsubo cardiomyopathy in viral infections include catecholamine-induced myocardial toxicity and inflammation related to sepsis. Differential diagnoses include myocarditis and myocardial infarction. Evidence of the benefit of immunomodulatory drugs and dexamethasone are growing to support this hypothesis in COVID-19.

## Introduction

The outbreak of novel coronavirus disease 2019 (Covid-19) spread worldwide since the end of 2019. Takotsubo cardiomyopathy is a well-described reversible myocardial dysfunction, triggered by emotional or physical stress. Previously described in viral infections, causal mechanisms remain unclear between direct viral cardiac injury and secondary inflammation. Consequently, Takotsubo cardiomyopathies have been described in Covid-19 patients (1), and hereafter, we describe one such case, to discuss plausible mechanisms and management of these potentially severe occurrences.

## Case Report

A 79-years-old man presented to the emergency department for fever, cough, and increasing dyspnea. Previous medical history included hypertension, type 2 diabetes, and chronic kidney disease (estimated baseline glomerular filtration rate 59 ml/min), without any history of cardiac complication due to his cardiovascular risk factors. He had been symptomatic for a week and treated with cefpodoxime for 5 days.

**Figure 1:**
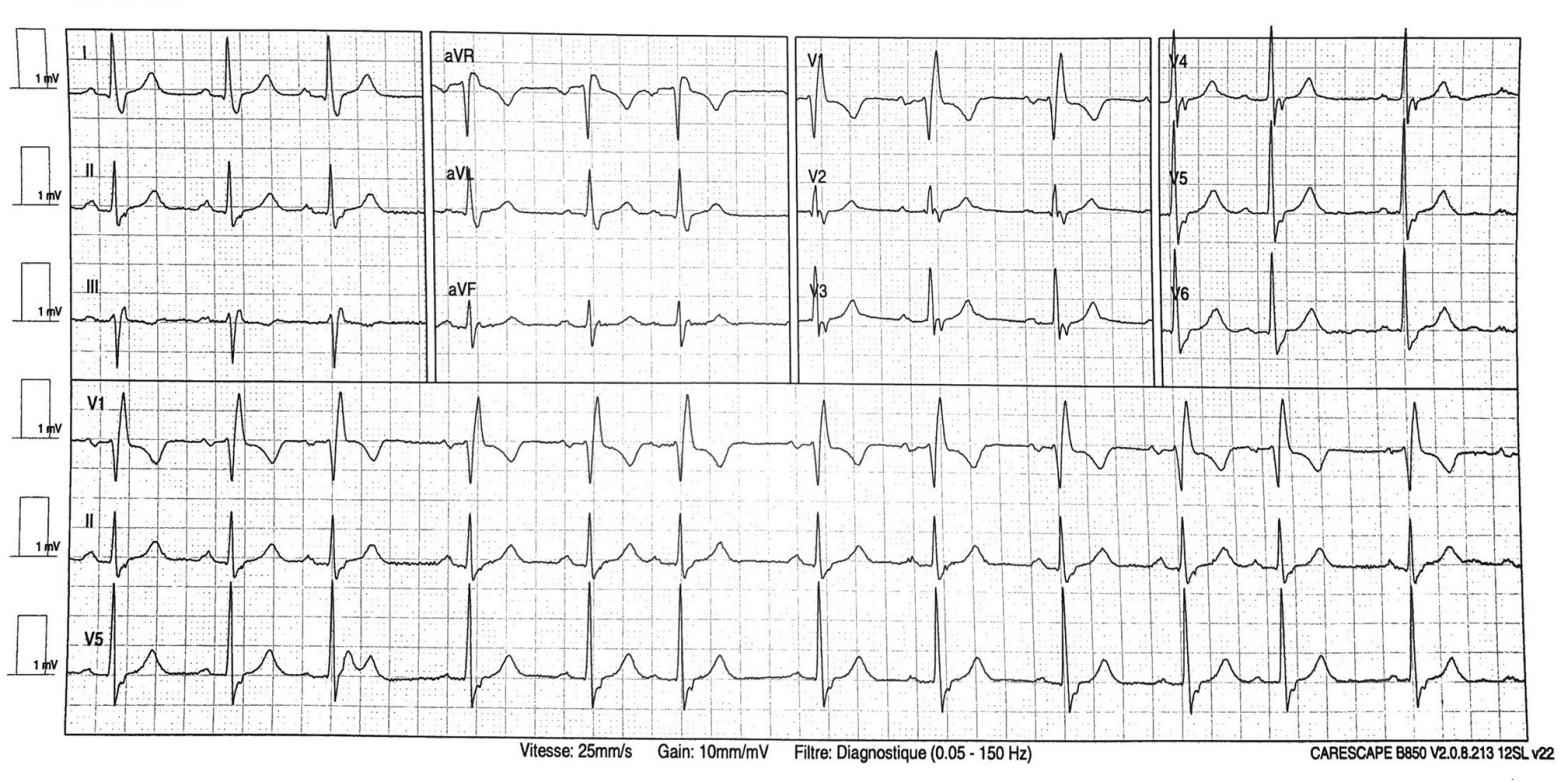
Electrocardiogram at admission.

**Figure 2:**
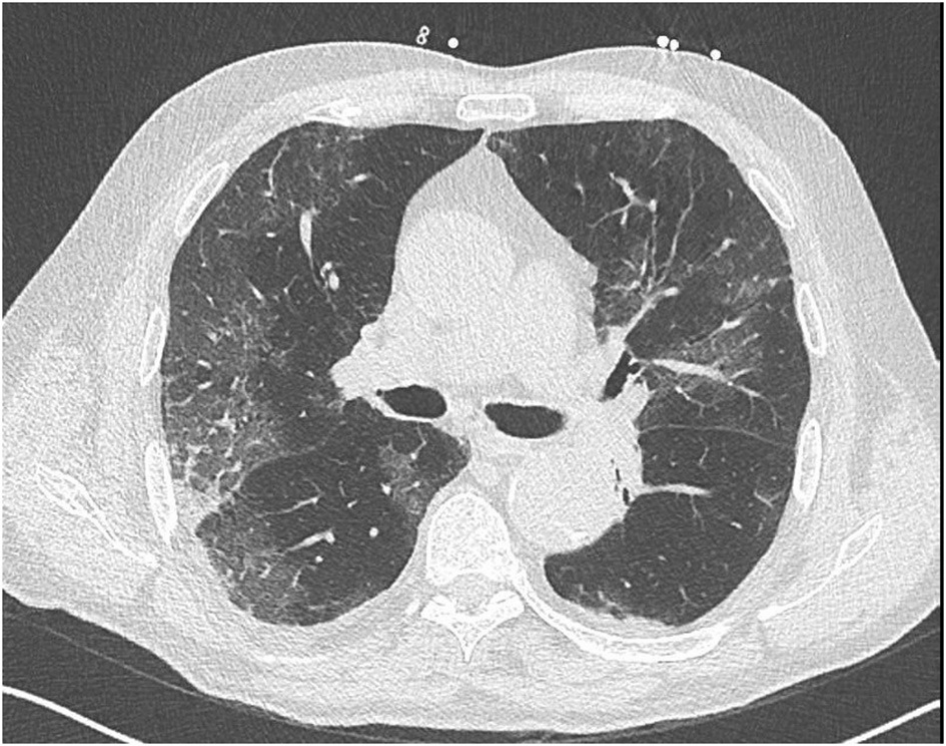
Computed tomography lung scan at baseline.

At admission, he presented talking dyspnea, tachypnea (respiratory rate of 24 cycles/min), low pulse oxygen saturation (SpO_2_ 93%), and bilateral diffuse crackling. He required 3 l/min nasal O_2_ support. He showed no fever (temperature 37.2°C). Electrocardiogram (ECG) showed sinus rhythm with neither conduction nor repolarization disorder (see Figure 1). Lung computed tomography scan showed typical bilateral opacity suggesting severe acute respiratory syndrome coronavirus 2 (SARS-Cov-2) infection (see Figure 2). Nasopharyngeal polymerase chain reaction confirmed diagnosis of SARS-Cov-2 infection. Present at baseline were moderate lymphopenia (1.33 g/l) and inflammatory syndrome (fibrinogen 7.88 g/l, ferritin 665 μg/l, interleukin-6 520 pg/ml, C-reactive protein 339.4 mg/l, procalcitonin 4.97 ng/ml, and neutrophil count 7.95 g/l). Creatinine was elevated (197 μmol/l) corresponding to an estimated glomerular filtration rate of 30 ml/min/1.73 m^2^. Other lab results were normal (troponin was not assayed at admission).

**Figure 3:**
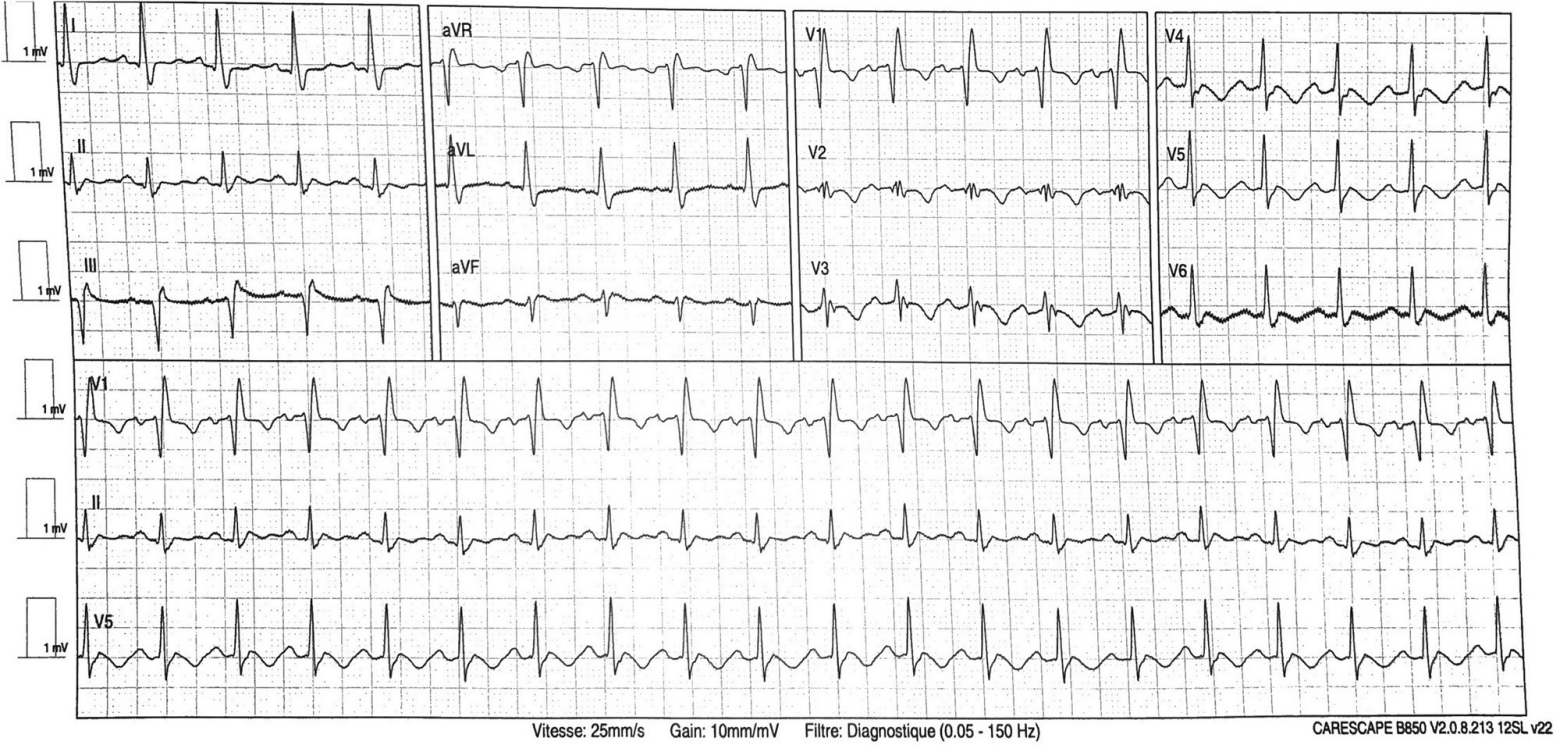
Electrocardiogram on day 2, during circulatory failure.

The patient quickly deteriorated and required transfer to the intensive care medicine department for acute respiratory failure within the same day. He was supported by mechanical ventilation with sedation and neuromuscular blocking agents. Wide-spectrum antibiotic therapy with cefotaxime 6 g per day and rovamycin 9 million UI per day for 5 days was administered due to suspicion of bacterial coinfection, in association with lopinavir–ritonavir targeting COVID-19 (200 mg/50 mg per day). On day 2, he showed signs of circulatory failure (unassisted systolic arterial pressure 80 mmHg, metabolic acidosis with pH 7.28, and lactate 2.1 mmol/l) with acute kidney injury requiring catecholamine support by norepinephrine (up to 1.04 μg/kg/min). ECG showed non-elevated ST segment, prolonged QT interval, T wave inversion (see Figure 3), and increased highly sensitive troponin T (up to 637 ng/l). Transthoracic echography was inconclusive due to poor echogenicity and was completed by transesophageal echocardiography, which showed left ventricular failure with reduced ejection fraction (LVEF 40%) and typical apical ballooning suggesting Takotsubo cardiomyopathy (see Supplementary Videos 1, 2). Coronary angiography was discussed; however, troponin spontaneously decreased within 24 h to 433 ng/l and follow-up echocardiography showed restoration of LVEF with decrease of apical ballooning aspect. ECG anomalies with T wave inversion persisted afterwards. Circulatory failure resolved within 2 days allowing catecholamine weaning. However, patient presented refractory acute respiratory distress syndrome and acute kidney injury requiring hemodialysis. Before cardiac magnetic resonance imagery (cMRI) could be performed, patient died 13 days later due to septic shock with multi-organ failure, secondary to ventilator-acquired pneumonia. Cardiac involvement was excluded as cardiac index was elevated (3 l/min/m^2^) and Takotsubo cardiomyopathy was ruled out by transesophageal echocardiography. A timeline summarizing these events is presented in the Supplementary Material.

## Discussion

Takotsubo cardiomyopathy is an acute heart failure syndrome with specific dyskinetic abnormality, depicted after the traditional Japanese octopus-trap (2). Although described since 1990, various definitions still co-exist (see summary of diagnostic criteria in Table 1). The latest, described in the 2018 European Society of Cardiology Expert Consensus Document on Takotsubo cardiomyopathies listed criteria, required to assert this diagnosis (3). In the present case, the computation of the InterTAK diagnostic score yielded a total above 70 points, corresponding to a high probability of Takotsubo. Following the diagnostic algorithm, ECG was in favor with a lack of ST-segment elevation or depression, and QT interval was indeed prolonged. Echocardiography showed typical Takotsubo cardiomyopathy with circumferential wall motion abnormalities and apical ballooning, and left ventricular outflow tract obstruction, mitral regurgitation, and right ventricular failure were excluded. Patient being unstable, coronary computed tomography angiography was precluded. Coronary angiography was discussed; however, three elements prevented us to perform this exam: (i) patient was too unstable to be transported (requiring high doses of vasopressors and heavy oxygenation support due to severe acute respiratory distress syndrome), (ii) circumferential wall motion abnormalities could not be explained by a single coronary artery obstruction, and (iii) patient was already treated by adequate antithrombotic treatments. It must be further noted that, if performed, a coronary angiography may yield significant coronary artery disease; however, the presence of significant lesions do not exclude a diagnosis of Takotsubo cardiomyopathy; in this case, these lesions do not explain the observed regional wall motion abnormalities, which were circumferential (3). The resolution of these abnormalities with troponin and inflammatory biomarker decrease and restoration of LVEF and wall motion comforted this choice. Finally, at the time of caring for this patient, routinely performing coronary angiography in patients with COVID-19 was not easy due to safety risk for healthcare personnel not trained for viral outbreaks, a feature made easier since then (4).

**Table 1:** Takotsubo diagnosis criteria, according to Mayo Clinic, European Society of Cardiology (ESC), and Heart Failure Association (HFA).

	**Mayo Clinic**	**ESC, InterTAK criteria**	**HFA criteria**
Echocardiography	Transient regional wall motion alteration	Apical ballooning Right ventricular involvement	Transient regional wall motion alteration
Coronary angiography	Absence of coronary artery disease which may explain the observed wall motion abnormalities	Possible coronary artery disease	Absence of coronary artery disease which may explain the observed wall motion abnormalities
Electrocardiogram (ECG)	New ECG repolarization abnormalities	New ECG repolarization abnormalities are present Possible no ECG changes	New and reversible repolarization ECG abnormalities
Cardiac Biomarkers	Modest elevation troponin	Modest troponin or brain natriuretic peptide elevation	Natriuretic peptide or troponin elevation
Differential diagnosis	Pheochromocytoma or Myocarditis	Infectious myocarditis	
Trigger	Possible stress trigger	Possible emotional, physical (neurologic disorders or pheochromocytoma) or combined Predominantly post-menopausal women	Possible stressful trigger

Although traditionally associated with psychological or physical stress, cases have been reported during viral sepsis (5) and most recently in COVID-19 (6, 7). A case series reported by Giustino et al. described five cases of Takotsubo cardiomyopathy, out of 118 consecutive patients (4.2%) with COVID-19 who underwent transthoracic echocardiography exploration, with similar reported resolution of echocardiographic features (8).

Mechanisms are plural and include catecholamine-induced myocardial toxicity and inflammation related to sepsis, which may be intertwined.

Catecholamine-induced cardiotoxicity may be associated with the visual aspect of apical ballooning with relative hypokinesia, due to the distribution of β2 adreno-receptors more prevalent in the apex (9). Indeed, myocardial beta-adrenergic toxicity is related to intra-cellular calcium dysregulation.

The sarco/endoplasmic reticulum Ca^2+^-ATPase (SERCa) is key to calcium homeostasis in the myocardium, by regulating excitation/contraction coupling via calcium distribution around the sarcoplasmic reticulum. Its inhibition is associated with acute heart failure (10). This inhibition may be triggered by (i) sarcolipin protein, overexpressed during events such as inflammation, leading to a decrease in its calcium affinity (11) and (ii) phospholamban protein lack of phosphorylation that maintains SERCa inhibition. In the present case, the patient required high-dose norepinephrine during septic shock combined with acute heart failure. However, given the more pronounced beta-adrenergic effect of dobutamine, as compared to norepinephrine, dobutamine was not administrated to prevent further toxicity.

Added to the beta-adrenergic toxicity with SERCa inhibition, catecholamine storms have been associated with microcirculatory dysfunction due to diffuse vasoconstriction. A series of Takotsubo biopsies showed microvascular endothelial cells apoptosis. Reported histology described contraction band necrosis, hypercontracted sarcomeres, dense eosinophilic bands, and interstitial mononuclear infiltration (12). Furthering the microvascular injury hypothesis, stress microRNAs including endothelin-1 were associated with myocardial ischemia during Takotsubo cardiomyopathy (13). In COVID-19 infections, the prevalence of non-obstructive acute myocardial injury was reported elevated. Possible associated mechanisms include septic microvascular dysfunction with endothelial abnormalities, destabilization of atherosclerotic plaques, and hypoxic injury (14). In one case of Takotsubo cardiomyopathy related to COVID-19, endomyocardial biopsy showed diffuse T-lymphocytic inflammatory infiltrates with increased CD3 cell count (15). It must be noted, however, that endomyocardial biopsies are not required to confirm this diagnosis, all the more so in unstable patients (3).

While myocarditis and Takotsubo cardiomyopathy share common mechanisms, in the latter, beta-adrenergic cardiotoxicity seems prevalent, with a synergistic effect of inflammation. In SARS-Cov-2, a minor form of cytokine-release syndrome (CRS) has been related to the increased activation of effector T cells and their production of high tumor necrosis factor (TNF) α, cytokine interleukin (IL)-6, IL-8, and chemokine ligand 1 (CXCL-1) level. These cytokines showed direct cardiac toxicity with negative inotropic effect and cell apoptosis associated with myocardial macrophage infiltration (16). In experimental models of CRS, catecholamines have been associated with immune dysregulation, through a self-amplifying loop in macrophages (17). In these models, atrial natriuretic peptides decreased catecholamine levels and, consequently, myeloid-derived cytokines including IL-1β, IL-6, and TNF. Because of this interplay between catecholamines and inflammation, both mechanisms may be involved in the genesis of Takotsubo cardiomyopathies in patients presenting with COVID-19 pneumonia. In our case, the patient presented elevated IL-6, which may give some substance to this hypothesis. As of yet, dexamethasone is one of the few molecules that showed unanimous efficacy in treating severe COVID-19 pneumonia, after the landmark Randomized Evaluation of COVID-19 Therapy (RECOVERY) trial (18). Likewise, other immunomodulatory molecules have been tested in these indications, however with less success, such as the Janus kinase inhibitor, baricitinib (19), and the IL-6 inhibitor, tocilizumab (20).

## Conclusion

COVID-19 may be associated with Takotsubo cardiomyopathy in the context of marked inflammatory syndrome, and reasoned use of catecholamines should be invoked whenever feasible, due to a plausible interplay between inflammation and catecholamines. Diagnostic algorithm may include coronary angiography; however, the presence of coronary lesions does not exclude a diagnosis of Takotsubo cardiomyopathy, if the observed regional motion wall abnormalities are not explained by the lesions.

## Data Availability Statement

The original contributions presented in the study are included in the article/Supplementary Material, further inquiries can be directed to the corresponding author/s.

## Ethics Statement

Written informed consent was obtained from the individual(s) for the publication of any potentially identifiable images or data included in this article.

## Author Contributions

SO wrote the initial draft. MJ and J-PM provided critical review to the manuscript. LN supervised this work and wrote the final manuscript. All authors contributed to the article and approved the submitted version.

## Conflict of Interest

The authors declare that the research was conducted in the absence of any commercial or financial relationships that could be construed as a potential conflict of interest.

## Supplementary Material

Supplementary Video 1 Transesophageal echocardiography three-chamber view on day 2.

Supplementary Video 2 Transesophageal echocardiography two-chamber view on day 2.

Supplementary Table 1 Events timeline.
