# Supplementary material for: Case Report: Takotsubo Syndrome Associated With Novel Coronavirus Disease 2019
Source: Front Cardiovasc Med. 2021 Feb 22;8:614562. doi: 10.3389/fcvm.2021.614562 (PMC7937625; doi:10.3389/fcvm.2021.614562)
Supplement: Supplementary Table 1 — Events timeline. [file Data_Sheet_1.PDF]

Supplementary Table. Events timeline.

Time	Events
Day 0	First symptoms: fever, cough and increasing dyspnea
Day 1	Introduction of cefpodoxime for 5 days by general practitioner
Day 7	10:00 Emergency room examination with hypoxia (SpO_2 93% requiring oxygen support 3 L/min). Diagnosis of SARS-CoV-2 infection by RT-PCT made on a nasopharyngeal swab sample. ECG showed regular sinus rythm without repolarization disorder
	14:00 Rapid respiratory deterioration requiring transfer in intensive care department
	15:00 Mechanical ventilation under sedation, wide spectrum antibiotics (cefotaxime and rovamycin), lopinavir-ritonavir
Day 9	Circulatory failure with lactate 2.2 mmol/L, need for norepinephrine (up to 1.04 µg/kg/min). EKG showed diffuse T wave inversion and QT interval prolongation. Troponin T levels elevated to 637 ng/L. Echocardiography showed LVEF decrease and LV apical ballooning akinesia and relative hyperkinetic basal contraction.
Day 10	Spontaneous decrease of troponin T to 433 ng/L.
Day 13	Restoration of left ventricular function and decrease of the Tako-Tsubo features.
Day 22	Died of multiorgan failure (septic shock, acute kidney injury, acute respiratory distress syndrome). Cardiac index was normal with 3 L/min/m²

Abbrevations : LV: left ventricle ; LVEF: left ventricular ejection fraction ; RT-PCR: reverse-transcriptase polymerase chain reaction; SARS-Cov-2: severe acute respiratory syndrome due to coronavirus-2; SpO_2 : pulse oxygen saturation
